# Why Are Male Social Relationships Complex in the Doubtful Sound Bottlenose Dolphin Population?

**DOI:** 10.1371/journal.pone.0000348

**Published:** 2007-04-04

**Authors:** David Lusseau

**Affiliations:** 1 University of Otago, Department of Zoology, Dunedin, New Zealand; 2 Dalhousie University, Department of Biology, Halifax, Nova Scotia, Canada; Emory University, United States of America

## Abstract

**Background:**

Access to oestrus females tends to be the main driver of male sociality. This factor can lead to complex behavioural interactions between males and groups of males. Male bottlenose dolphins may form alliances to consort females and to compete with other males. In some populations these alliances may form temporary coalitions when competing for females. I examined the role of dyadic and group interactions in the association patterns of male bottlenose dolphins in Doubtful Sound, New Zealand. There is no apparent mating competition in this population and no consortship has been observed, yet agonistic interactions between males occur regularly.

**Methodology/Principal Findings:**

By comparing the network of male interactions in several social dimensions (affiliative, agonistic, and associative) I show that while agonistic interactions relate to dyadic association patterns, affiliative interactions seem to relate to group association patterns. Some evidence suggests that groups of males also formed temporary coalitions during agonistic interactions. While different groups of males had similar relationships with non-oestrus females, the time they spent with oestrus females and mothers of newborns differed greatly.

**Conclusions/Significance:**

After considering several hypotheses, I propose that the evolution of these complex relationships was driven by sexual competition probably to out-compete other males for female choice.

## Introduction

The sociality of males tends to be driven by access to opportunities for reproduction [1]–[3]. Complex social behaviour may evolve that allows individual males to maximise either access to females or their inclusive fitness, especially in slowly reproducing species [1]. These behaviours can vary widely in diversity as well as in complexity and form networks of relationships that can in turn influence one another. Cognitively complex species can for example engage not only in agonistic (fighting, threat displays…) and affiliative behaviour (grooming, proximity…), but also in behaviour emerging from the interactions between these social dimensions, such as reconciliation, retribution, or policing [4], [5]. For example, agonistic interactions between two individuals will influence how they interact in their proximity network (they might start spending less time together) or their grooming network (they might increase grooming after fights to reconcile).

More importantly, studies of bottlenose dolphins and nonhuman primates suggest that both individual- and group-level processes can influence the evolution of these networks [6]–[12]. For example male primates will form alliances and coalitions to defend or acquire resources [7] or may carry out affiliative behaviour to maintain bonds within groups [12]. However, individual-level processes such as dominance ranking also play a role in the dynamics of male primate interactions [1]. It becomes apparent that to understand fully the nature of social relationships between individuals it is necessary to integrate information from the different social realms in which they interact.

Male bottlenose dolphins (*Tursiops* spp.) form complex social bounds that can last decades [6]. Alliances have been observed in Shark Bay, Australia, where male dolphins require allies to consort females. There, individuals that are often seen associated together will co-operate to attack other male alliances or to separate a female from her group. Alliances also form temporary coalitions, for the same purpose. Alliances have also been observed in Port Stephens, Australia [13], Sarasota Bay, Florida [14], and in the Bahamas [15]. Interestingly the choice of allies seems to be related to kinship in some populations [15] but not others [13], and the role of kinship in ally selection can even vary within a population [16]

The behaviours used to mediate competition and to maintain these affiliations are poorly understood in cetaceans. I describe here the pattern of male-male social interactions using two behavioural proxies and their relationship to their association patterns in the bottlenose dolphin population of Doubtful Sound, New Zealand [17], [18]. This study aims to relate behavioural interactions in agonistic and affiliative settings to the observed association patterns, defined as individuals being present together in a school, in order to understand the relationships of both individuals and groups of individuals in different social networks.

Dolphins spend most of their time underwater and therefore the directionality of behaviour, as well as the number of individuals involved in interaction bouts can be misjudged if observing only from the water surface. I present here observations of two particular behavioural events that circumvent this problem because they can be completely observed from the water surface and they involve two individuals at a time (in rare occasions three individuals can be involved). The first event, headbutting, occurs during agonistic interactions between males [19]–[22]. The second one, mirroring, also occurs when males interact socially. Mirroring is a form of non-agonistic and non-sexual physical contact between males and is similar to the petting behaviour described in Shark Bay, Australia, or off Mikura Island, Tokyo, Japan, which represents a form of affiliative behaviour [23]–[26].

The Doubtful Sound population is small (60–65 individuals at the time of the study) and essentially closed to immigration and emigration (Lusseau et al. 2003; Williams et al. 1993). The adult population at the time of the study was composed of 49 individuals that had all been sexed (26 males and 23 females) which lived in multi-male, multi-female schools [17]. The reproductive output of the population is low [27] which should increase competition for mating access. Yet, neither female consortship, nor direct competition to mate with females, nor infanticides has been observed. This might indicate a less complex mating strategy which should relax the need to maintain alliances and higher-order relationships in this population [1], [2]. Yet long-term relationships between males still exist [17]. Here I therefore test whether group-level processes, such as instances of coalition formation, are taking place in the dynamics of male-male relationships. I also quantify the role of individual and group processes in the observed patterns of interactions. I finally assess whether mate access may play a role in the evolution of these relationships despite the lack of apparent direct competition.

## Results

**Figure 1:**
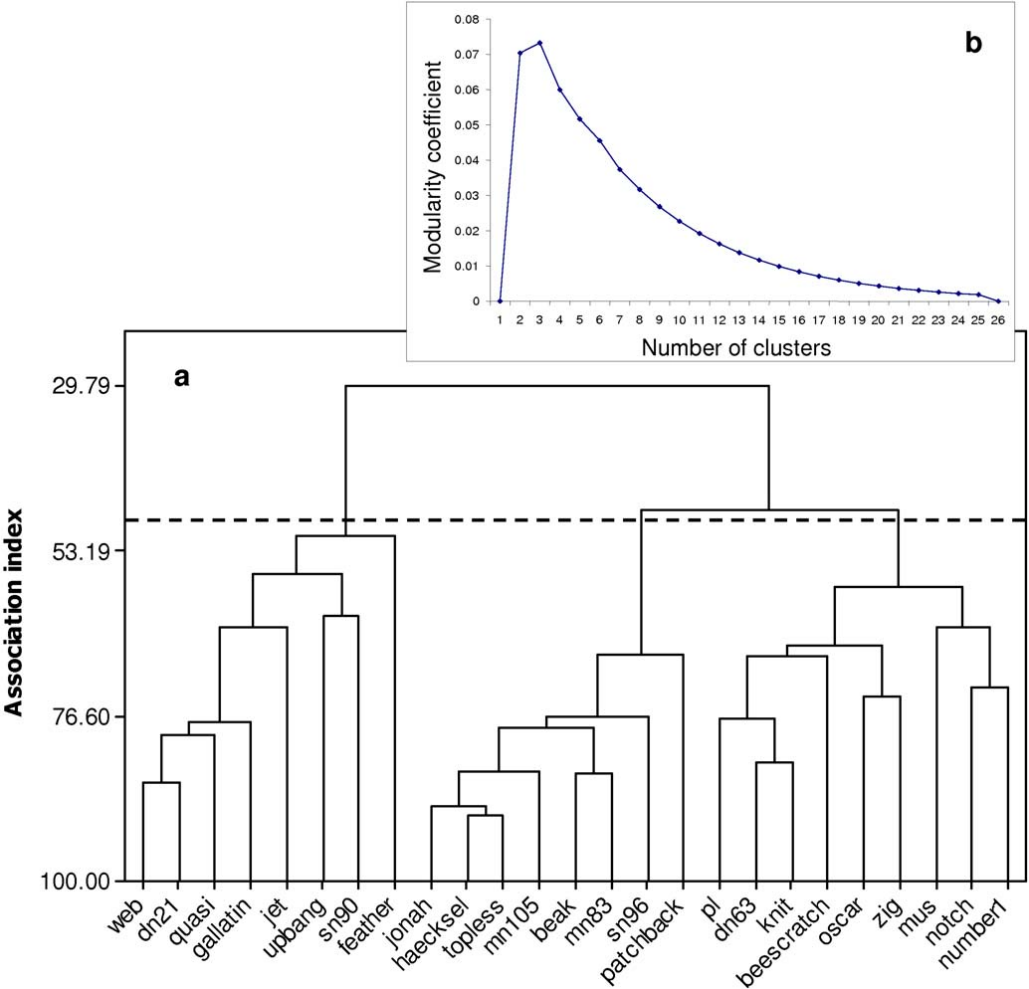
(a) Average linkage cluster analysis of the male association matrix, based on half-weight index (the association index). There appears to be three clusters of individuals spending more time together, the dashed line represents the more parsimonious split in the network which is given by the peak in the modularity coefficient (b).

**Figure 2:**
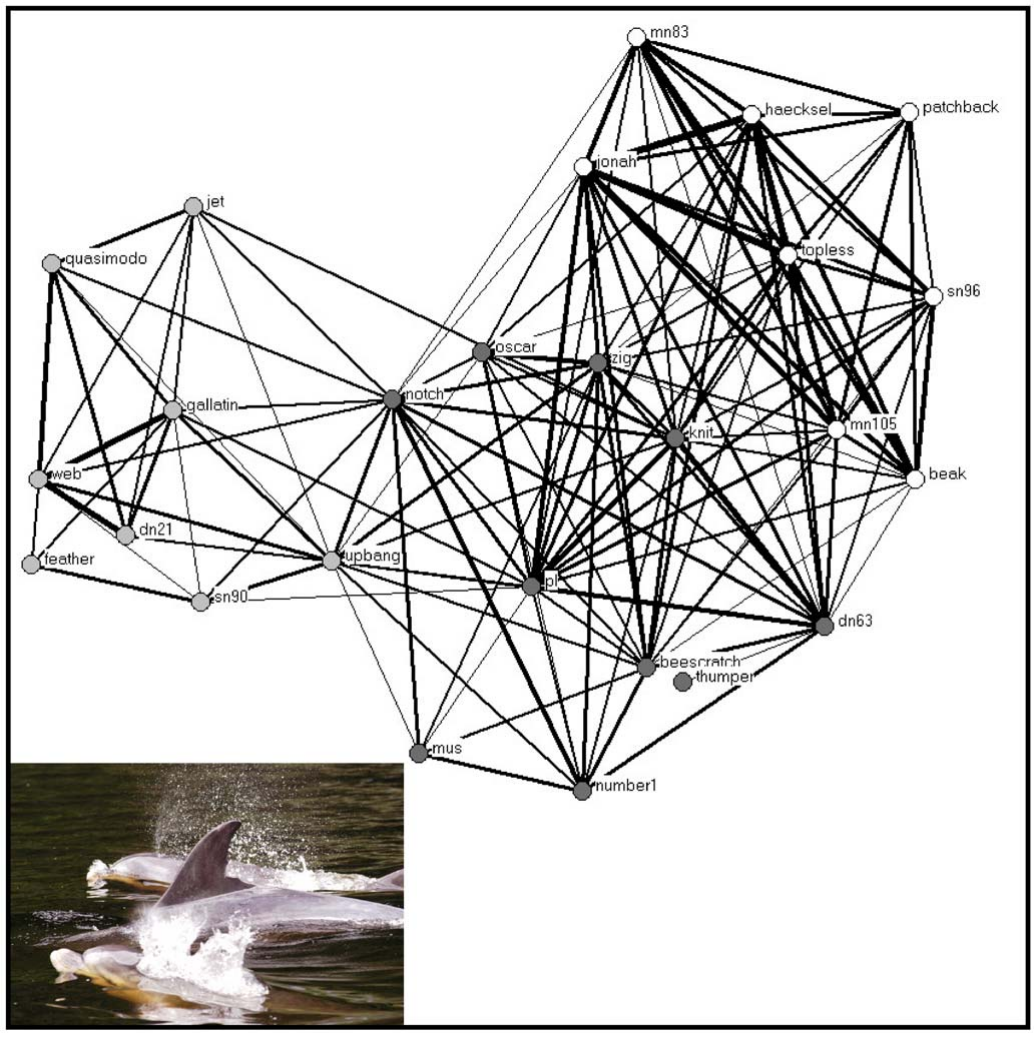
Social networks of male bottlenose dolphin interactions in Doubtful Sound, New Zealand using association to define relationships (Half-weight index, only dyads with HWI>0.50 are represented). The thickness of the lines represents the strength of association. The colour of the vertices represents the three clusters identified in Figure 1.

**Figure 3:**
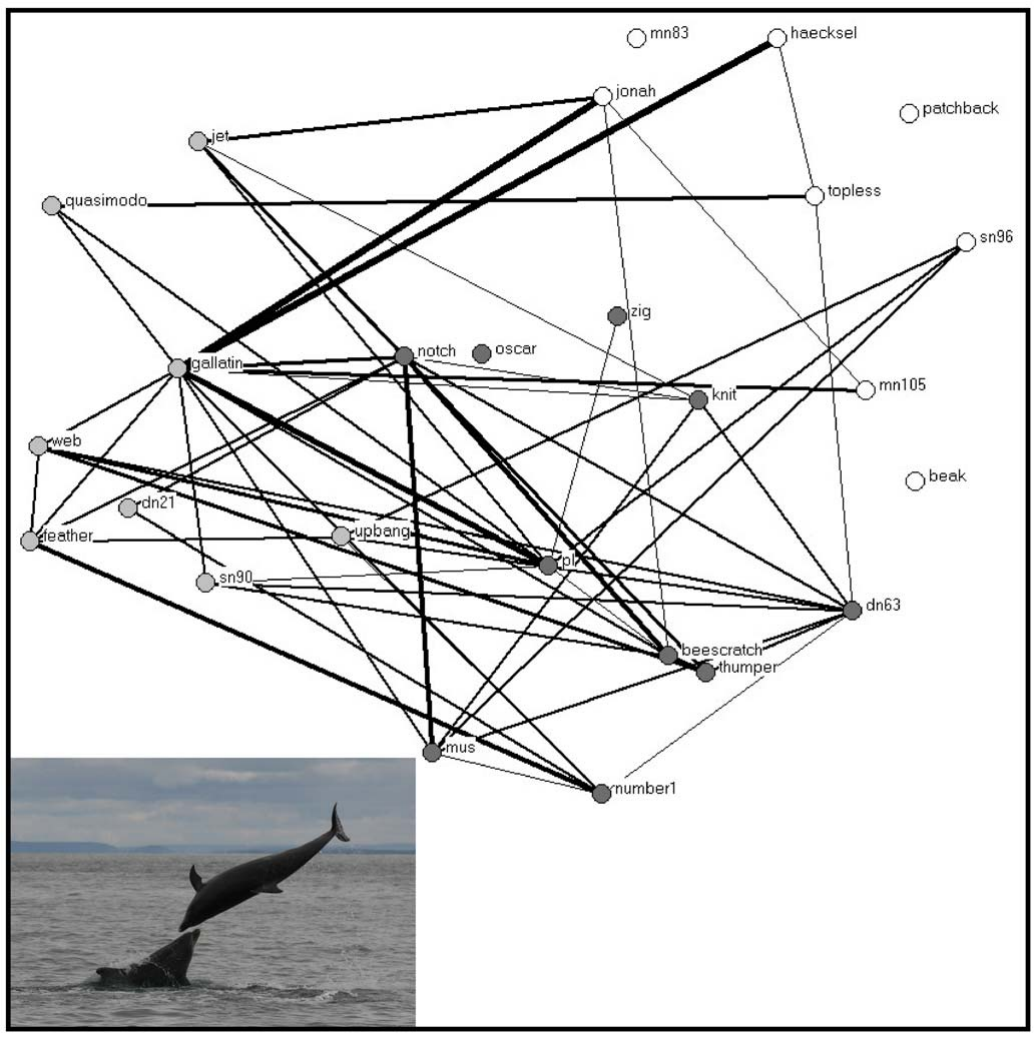
Social networks of male bottlenose dolphin interactions in Doubtful Sound, New Zealand using headbutting rate to define relationships. The thickness of the lines represents the number of behavioural bouts observed. Numbers of headbutting were standardized to the number of focal follows in which both animals were observed together. The colour of the vertices represents the three clusters identified in Figure 1.

**Figure 4:**
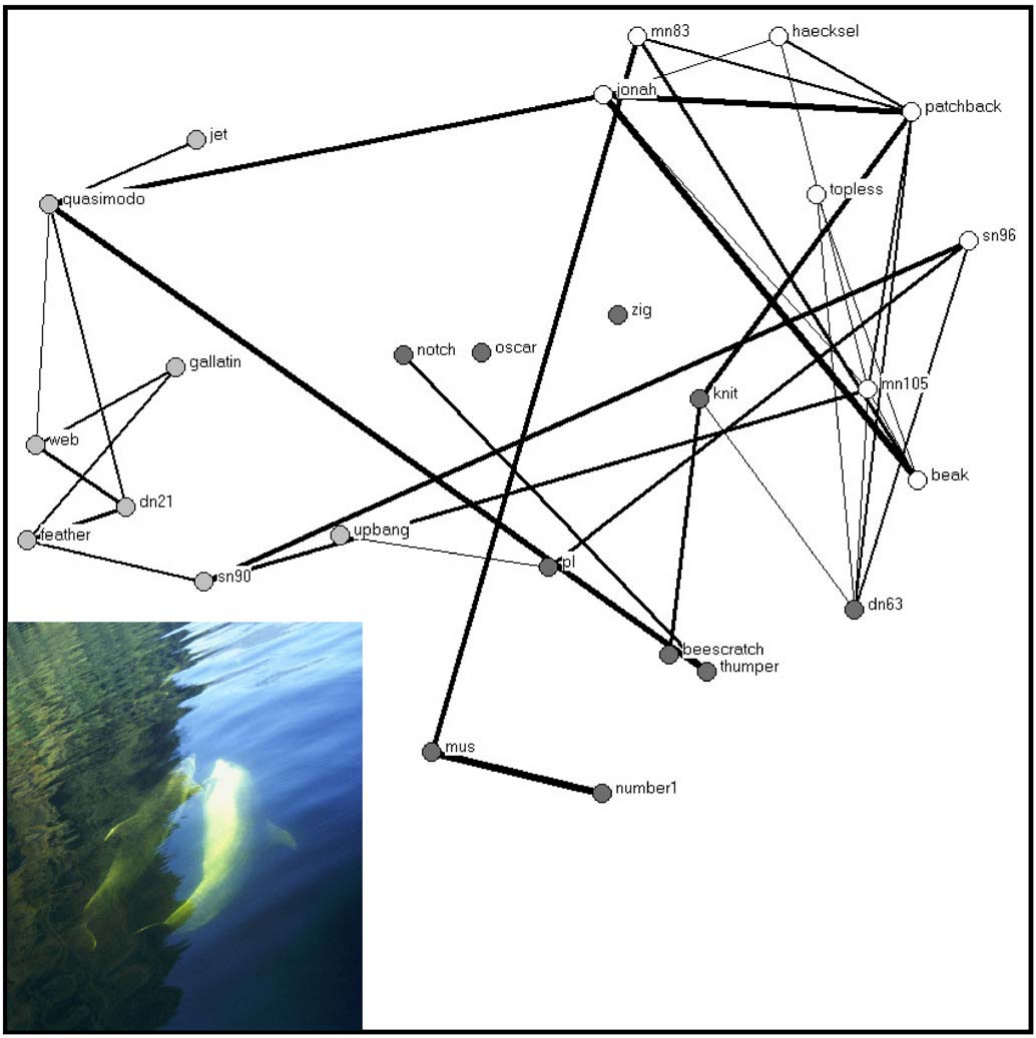
Social networks of male bottlenose dolphin interactions in Doubtful Sound, New Zealand using mirroring rate to define relationships. The thickness of the lines represents the number of behavioural bouts observed. Numbers of mirroring were standardized to the number of focal follows in which both animals were observed together. The colour of the vertices represents the three clusters identified in Figure 1.

The membership of 362 schools was identified during the study period. School members were defined as associated [28]. The more parsimonious clustering step for the association matrix segregated males into three groups (Figure 1). These will be defined as the Jonah, Web, and PL groups thereafter (Figure 2). A total of 73 headbutting (Figure 3) and 47 male-male mirroring bouts (Figure 4) were observed in which both participants were identified.

### Relationships between the social networks

The headbutting network was inversely correlated with the association network (Mantel test: r = −0.46, p<0.0001) while the mirroring matrix was not (Mantel test: r = 0.20, p = 0.08). That is, individuals were more likely to be observed headbutting with males with whom they spend less time, but the amount of time two individuals spent together did not influence the likelihood these two individuals would be mirroring. I used a partial mantel test to control for the association index matrix when comparing headbutting and mirroring to association at the group level. This technique helped understanding the relative importance of the groups in the occurrence of behaviours given the amount of time pairs of individuals spent together. I find that while individuals were more likely to engage in mirroring with individuals from the same group (r = 0.39, p = 0.011) they were not more likely to headbutt with individuals based on their group membership (r = 0.02, p = 0.436). Therefore while headbutting occurrence is related to pairwise association, mirroring occurrence relates to social group membership.

### Network analyses

There was no relationship between centrality measures in the association and mirroring networks, therefore the position of individuals in the social network did not relate to its involvement in affiliative interactions. Strength in association network was not related to centrality measures in the headbutting network. Hence the position of individuals within the association social network did not influence their position in the agonistic social network.

The clustering coefficient of individuals in the association network was negatively related to their strength (F_1,25_ = 6.9, p = 0.015, r^2^ = 0.25) and their degree (F_1,25_ = 8.9, p = 0.006, r^2^ = 0.27) in the headbutting network. It was not related to the number of headbutt they performed within their group (F_1,25_ = 2.5, p = 0.13, r^2^ = 0.09), but was negatively related to the number of headbutt they were involved in with members of other groups (F_1,25_ = 11.8, p = 0.002, r^2^ = 0.33). Thus, the more individuals had a tightly-knitted group of associates, the less likely that individual was to be involved in agonistic interactions with males from outside its group.

### Triadic agonistic interactions

On five occasions I was able to identify all opponents of headbutting bouts involving three individuals (Table 1). All coalitions were formed of individuals from the same school. In two cases the two individuals that joined forces to fight another were from the same group but were not immediate associates (Gallatin and Jet against Jonah, Gallatin and SN90 against PL, Figures 1 and 2). In the three remaining instances the two allied individuals were pairs that were identified as spending the most time together (pairs with the highest association index for each individual involved in the pair).

**Table 1:** Identity of individuals involved in triadic headbutting bouts.

allied males	opponent
Gallatin and Jet	Jonah
Gallatin and SN90	PL
Jonah and Haecksel	Gallatin
DN63 and Knit	Notch
DN63 and Knit	PL

The left column represents the two males allied against the individual in the right column. Refer to Figure 1 for the associative relationship among these males.

### Relationships with females

**Figure 5:**
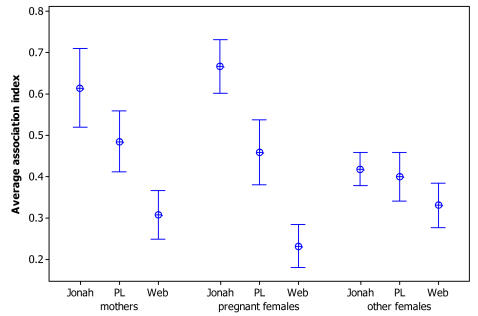
Average association index between members of each male group and 3 types of females in the population: pregnant, new mothers, and others. Symbols are means and bars are 95% confidence intervals.

The three male groups did not differ in their association rate with females that either had no calves or had older calves (Figure 5, F_2,51_ = 3.1, p = 0.06) even though there was a non-significant trend for the Jonah group to have higher association rate with those females than the Web group did. However they differed in their associations with new mothers (Figure 5, F_2,51_ = 16.5, p<0.001), and with oestrus females (Figure 5, F_2,51_ = 41.7, p<0.001). The Jonah group spent significantly more time with oestrus females than the other groups (Figure 5), and the Jonah and PL groups spent significantly more time with new mothers than the Web group (Figure 5). Also, the group membership effect size decreased from oestrus females (largest difference in means between Jonah and Web groups, 0.43, SE = 0.048) to new mothers (largest difference in means between Jonah and Web groups, 0.31, SE = 0.054).

## DISCUSSION

Male bottlenose dolphins in Doubtful Sound engaged primarily in dyadic interactions during agonistic interactions and in higher-order relationships in affiliative contexts. The association network was composed of three groups of individuals. Agonistic interactions depended on the time individual spend together and they could occur both within and between these groups. In addition, observations of triadic headbutting bouts show that individuals were able to rely on short-term coalition formation with other members of their group during agonistic interactions. This result is corroborated by the fact that males which had more closely related associates, defined by a higher clustering coefficient, tended to be exposed to less agonistic interactions. Affiliative behaviour tended to occur more within groups of males than between groups. Despite being a group-related behaviour, some mirroring occurred between individuals from different groups, showing the dynamic nature of relationships as highlighted by previous analyses [18], [29].

These relationships between the different male social networks stress that interactions are governed by both individual- and group- level processes. Male relationships in this population are therefore not only driven by individual needs but also by the maintenance of bounds with a pool of potential allies. Individuals can not only rely on these associates during agonistic interactions, but also the social relatedness of those social group co-members dictate the level of aggression to which an individual is exposed, providing benefits for the maintenance of these social groups. While this situation is not uncommon in cognitively-complex social mammals [6], [8], it usually arises from more intense competition for female access which is not apparent in the Doubtful Sound population.

However, it appeared that different groups have different association rate with oestrus females and new mothers. The PL group, which is composed of younger males [18], did not differ in the rate at which it associated with new mothers and oestrus females from the way it did with other females. The Jonah group however spent significantly more time with females in oestrus and new mothers than with other females while for the Web group the situation was reversed. There appears to be a discrepancy in the amount of time groups of males spent with oestrus females and it seems that this difference remains present to a lesser extent once these females give birth. In a small population which needs to live in large schools [17], consortship might require a longer involvement in mate guarding, which would be hard to be met by individuals or pairs of individuals. Being able to rely on a group of co-allies can reduce the cost of long-term mate guarding. While this situation is rare in birds and mammals, it has been reported in chimpanzees (*Pan troglodytes*) [8]. Alternatively, in a mating system driven by female selection being able to exclude other males from the vicinity of oestrus females means that individuals can be more readily picked as a favourite partner. Again higher-order association patterns would be helpful to diminish the cost of competition in a tit-for-tat scheme [30] or through kin selection [31]. The fact that there is no overt competition to mate with oestrus females favours this hypothesis over male mate guarding.

It is also possible that mating does not play a role in this discrepancy in association between individual males and females. Role specialisation is known to occur in bottlenose dolphin's hunting strategies, at least in some populations [32], and therefore it is possible that different individuals play a disproportionate role in hunting or predator defence. While this could explain a disproportionate preference for certain males by energetically-challenged females (pregnant or lactating), it does not explain why females in oestrous should prefer more these males compared to females in a non-reproductive stage. It is also possible that age plays an important role in the establishment and maintenance of inter-sexual relationships. It is therefore possible that cohorts of the same age would be more likely to stay together. However, there is no clear age distinction between reproducing females and others during the study period.

These hypotheses raise the issue of the relevance of kin selection in the evolution of male social relationships in this population. While kin selection would readily explain the benefits of coalitionary relationships to mate, either through mate guarding, or through competitor exclusion, it is not supported by the apparent male grouping pattern in which age seem to play a role [18]. As in other bottlenose dolphin and chimpanzee populations, social development within an age cohort may play a crucial role in the selection of associates in this population. There is support for mutualism in dolphins [33] which would provide a viable alternative to kin selection for the evolution of these male relationships [8], [12]. At this stage no genetic information is available about individuals in this population, but assessing male relatedness and paternity success would help test these hypotheses.

Male groups seem to also associate disproportionately with new mothers, which could be explained by infanticide risks. While Infanticide rate is either extremely low or non-existent, it does not mean that infanticide risk does not exist [1]. There is a direct incentive for males not related to the mother or father of the newborn to kill it in order to gain access to a female with a higher fitness to reproduce [34]. Continued association of male groups with new mothers may represent a form of allocare to prevent infanticide. It is also possible that some males are less prone to infanticide and hence are preferred companions for new mothers. Paternity testing would help in assessing these hypotheses.

## Materials and Methods

### Field techniques

From December 1999 to December 2001 I spent 123 days conducting systematic surveys looking for schools of bottlenose dolphins in Doubtful Sound. I spent 808 hours looking for dolphins and 625 hours with focal schools. A school was defined as a number of dolphins that operated in a coordinated fashion. Individuals in a school followed the same direction and were cohesive in their movement [17]. All members of a school were assumed associated. Once a school of dolphins was encountered, individuals were photo-identified using natural markings on their dorsal fins [35]. Individuals with distinct markings were additionally identified visually. The gender of dolphins was determined by direct observations of the genital area and by observation using an underwater video camera mounted on a pole [36].

Individuals involved in headbutting and mirroring bouts were identified visually *ad libitum* while following a focal school. An event was only subsequently used in the analyses if both individuals were identified. The likelihood of identifying any of the males in the population was the same. The dorsal fins of all males were highly marked by tooth rakes and nicks and all males were similarly conspicuous. Therefore even though not all bouts could be sampled, all individuals had an equal chance of being sampled. This sampling was therefore proportional to the likelihood they engaged in a headbutting or mirroring bout and the number of time they were encountered. Therefore it was necessary to standardise the likelihood that a pair of individuals engaged in a behavioural event to the number of times this pair was encountered together. The number of focal schools in which the male dyad was observed was used as an indicator of the amount of time these males spent together.

### Association pattern analyses

The half-weight index (HWI) was used to quantify the frequency of association among males [37]:
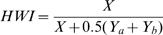
, where X is the number of schools where dolphin a and dolphin b were seen in the same school, Y_a_ is the number of sightings where dolphin a was sighted but not dolphin b, and Y_b_ is the number of sightings where dolphin b was sighted but not dolphin a. I used a hierarchical clustering analysis (average linkage measure) to determine whether groups of males could be segregated in the population. I used a modularity coefficient to define the more parsimonious clustering step as defined by the one providing a higher average association index within clusters and a lower average association index between clusters [38]. This modularity coefficient was extended to apply to weighted networks (eg, the association index matrix). At each step which divides the network into i clusters (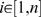) the modularity coefficient, Q, is estimated by summing the weight of associations for all dyads belonging to the same cluster, e_ii_, and compare it to what this summed weight would be if dyads associated at random in the network:


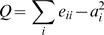
, where 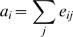 and e_ij_ is the sum of association indices linking individuals from cluster i to the ones of cluster j. This parsimonious coefficient had the advantage of not disregarding the possibility that all individuals belong to only one cluster.

### Network analyses

I calculated network statistics for individuals in the three social networks to determine whether the position of individuals in the association network influenced their position in the agonistic and affiliative networks. I first calculated the degree and the strength of individuals as a measure of their centrality in the networks. The degree is the number of links an individual has in a network. The strength provides additional information to the degree in considering not only the number of links an individual has, but also the weights of these links, in our case the association indices. So for a situation with m males the strength of individual *I*, s_i_ is:


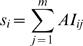
 where AI_ij_ is the association index between individual *i* and *j*.

Finally I also defined the grouping of individuals around an individual by calculating the local clustering coefficient. This provides a measure of how connected the associates of an individual are to one another. I use of modified version of the Barrat-Barthélemy clustering coefficient for this [39]:
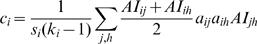



The first term of the equation is a normalisation term so that 0≤c_i_≤1; k_i_ is the degree of individual *i*; a_ik_ is 0 if AI_ik_ = 0 and 1 if AI_ik_>0. If there is no link between individual *j* and *h* then c_i_ is 0, if there is the contribution of the triad *i*,*j*,*h* to c_i_ is weighted by the association index between *j* and *h* (AI*_jh_*).

### Relationships with females

The breeding season is well defined in Doubtful Sound spanning from December to March, which corresponds to the austral summer [27]. Bottlenose dolphin gestation is roughly 12 months [40], therefore mating must take place during the same season. For each year I calculated the average association index between each male and three types of females: females that gave birth the following year (a conservative estimate of oestrus females), females that gave birth to a calf on that year (new mothers), and other females which could either have older calves or no calves.
